# Phosphorylation and Methylation of Proteasomal Proteins of the Haloarcheon *Haloferax volcanii*

**DOI:** 10.1155/2010/481725

**Published:** 2010-07-08

**Authors:** Matthew A. Humbard, Christopher J. Reuter, Kheir Zuobi-Hasona, Guangyin Zhou, Julie A. Maupin-Furlow

**Affiliations:** Department of Microbiology and Cell Science, University of Florida, Gainesville, FL 32611, USA

## Abstract

Proteasomes are composed of 20S core particles (CPs) of *α*- and *β*-type subunits that associate with regulatory particle AAA ATPases such as the proteasome-activating nucleotidase (PAN) complexes of archaea. In this study, the roles and additional sites of post-translational modification of proteasomes were investigated using the archaeon *Haloferax volcanii* as a model. Indicative of phosphorylation, phosphatase-sensitive isoforms of *α*1 and *α*2 were detected by 2-DE immunoblot. To map these and other potential sites of post-translational modification, proteasomes were purified and analyzed by tandem mass spectrometry (MS/MS). Using this approach, several phosphosites were mapped including *α*1 Thr147, *α*2 Thr13/Ser14 and PAN-A Ser340. Multiple methylation sites were also mapped to *α*1, thus, revealing a new type of proteasomal modification. Probing the biological role of *α*1 and PAN-A phosphorylation by site-directed mutagenesis revealed dominant negative phenotypes for cell viability and/or pigmentation for *α*1 variants including Thr147Ala, Thr158Ala and Ser58Ala. An *H. volcanii* Rio1p Ser/Thr kinase homolog was purified and shown to catalyze autophosphorylation and phosphotransfer to *α*1. The *α*1 variants in Thr and Ser residues that displayed dominant negative phenotypes were significantly reduced in their ability to accept phosphoryl groups from Rio1p, thus, providing an important link between cell physiology and proteasomal phosphorylation.

## 1. Introduction

Proteasomes are multicatalytic proteases found in all three domains of life and are essential for growth in many organisms, including haloarchaea and eukaryotes [1–5]. Accessory proteins, such as 19S cap and proteasome activating nucleotidase (PAN) complexes, and protein-modification pathways such as ubiquitination are important in regulating protein degradation by proteasomes. Proteasomal proteins are also subject to co- and posttranslational modification (PTM) including N-terminal acetylation [6–9], phosphorylation [10–27], S-glutathionation [28], N-myristoylation [29], O-linked glycosylation [30], and processing of N-terminal propeptides including exposure of the Thr active site residues of 20S core particles (CPs) [31].

Despite decades of study, the mechanisms and biological significance of many of the posttranslational modifications of proteasomes are poorly understood. In particular, our knowledge of the phosphorylation of proteasomes remains limited. Polo-kinase, casein kinase II and calcium/calmodulin-dependent protein kinase II are known to mediate the phosphorylation of 26S proteasomes in vitro [11, 13, 14, 16–19, 27, 32, 33]. There is also evidence that proteasomal phosphorylation is regulated by growth and/or signaling molecules such as *γ*-interferon [11, 12, 17, 22, 24, 26].

Our previous work demonstrated that archaeal proteasomes undergo a variety of PTMs and alterations in subunit composition. In *H. volcanii*, these PTMs include N-terminal acetylation of the CP *α*1 and *α*2, removal of the CP *β*-propeptide and phosphorylation of the CP *β* Ser129 [34, 35]. The subunit composition of *H. volcanii* proteasomal complexes also appears to change as cells enter stationary phase. *H. volcanii* synthesizes two different proteasome-activating nucleotidase proteins (PAN-A and PAN-B) and three different CP subunits (*α*1, *α*2, and *β*) [36, 37]. Proteasomal CPs and PAN complexes of different subunit composition have been isolated from *H. volcanii* ([36], this study and unpublished results). While the transcript levels encoding these proteasomal proteins all increase as cells enter stationary phase, only the protein levels of *α*2 and PAN-B increase several-fold during this transition [37]. Thus, the population of different proteasomal subtypes appears to be modulated by growth.

In this report, we elaborate on the PTM of *H. volcanii* proteasomal proteins including the mapping of additional sites of phosphorylation as well as a new type of modification for proteasomes, methyl-esterification. We also report a correlation between the phosphorylation state of the CP *α*1 protein and growth phase, provide evidence that Rio1p Ser/Thr kinase phosphorylates *α*1 and suggest through site-directed mutagenesis that the state of proteasomal phosphorylation influences cell physiology including viability and pigmentation.

## 2. Materials and Methods

### 2.1. Materials

Biochemicals were purchased from Sigma-Aldrich (St. Louis, MO). Other organic and inorganic analytical-grade chemicals were from Fisher Scientific (Atlanta, GA) and Bio-Rad (Hercules, CA). Radioactive chemicals were purchased from Perkin Elmer (Waltham, MA), and Strep chromatography resin was purchased from Qiagen (Valencia, CA). Desalted oligonucleotide primers were from Integrated DNA Technologies (Coralville, IA). Site-directed mutagenesis materials were from Invitrogen (Carlsbad, CA). Polyvinyl difluoride (PVDF) membranes for Western blots were from Amersham Biosciences (Piscataway, NJ). Alpha-casein was from ICN Biochemicals (Cleveland, OH).

### 2.2. Strains, Media, and Plasmids

Strains and plasmids used in this study are summarized in Supplementary Table 1 available online at doi:10.1155/2010/481725. *H. volcanii* strains H26 and GZ130 (Δ*psmA*) and *E. coli* strain BL21(DE3) were used as hosts for protein production and purification. *E. coli* strains were grown in Luria-Bertani medium at 37°C and 200 rpm, and *H. volcanii* strains were grown in ATCC 974 medium at 42°C and 200 rpm. Media were supplemented with 100 *μ*g/mL of ampicillin, 50 *μ*g/mL of kanamycin, or 0.1 *μ*g/mL of novobiocin as needed. For salt stress tests, the concentration of NaCl was reduced from the typical 2.25 M NaCl of the “normal” ATCC 974 medium to 1, 1.125, 1.25, 1.375, and 1.5 M NaCl. For the salt stress test inoculum, cells were grown in “normal salt” ATCC 974 medium by streaking cells from −80°C glycerol stocks onto fresh plates and growing these cells twice to log-phase in 2 mL medium. Each subculture was inoculated to a final OD_600_ of 0.01 to 0.02. Experiments were performed in biological triplicate and the mean ± S.D. was calculated.

### 2.3. Cloning and Site-Directed Mutagenesis

Site-directed mutagenesis (SDM) was performed using the Quikchange SDM kit as per manufacturer's instructions (Stratagene, La Jolla, CA) with the primers used for mutagenesis listed in Supplementary Table 2. The fidelity of all PCR-amplified products was confirmed by DNA sequencing (Genomics Core, Interdisiplinary Center for Biotechnology Research, University of Florida, Gainesville, FL) using the dideoxy termination method with Applied Biosystems (Foster City, CA) Model 3130 Genetic analyzer.

### 2.4. Protein Purification

Proteasomal proteins *α*1, *α*2, and *β* with C-terminal hexahistidine-(His_6_) tags were purified from recombinant *E. coli* BL21 (DE3) strains after induction isopropyl *β*-D-1-thiogalactopyranoside (IPTG) at 0.4 mM for 2 hours as previously described [36]. In addition, proteasomal proteins (*α*1, *α*2, *β*, PAN-A, and PAN-B) with C-terminal His_6_-tags, a Rio1p kinase homolog (HVO_0135) with a C-terminal -WSHPQFEK (-StrepII) tag and CPs composed of *α*1-His_6_ and *β*-StrepII were purified from recombinant *H. volcanii. *For protein purification, the various* H. volcanii* strains expressing these derivatives were grown to stationary phase (OD_600_ of 2.0–2.5), unless otherwise indicated. Phosphatase inhibitor cocktail I and II were included in all lysis buffers and diluted according to supplier (Sigma). For His_6_-tagged proteins, cells were lysed in 20 mM Tris pH 7.2 with 2 M NaCl (buffer A) supplemented with 5 mM imidazole. Cell lysate was loaded onto a nickel nitrilotriacetic acid (Ni-NTA) column (1.6 × 2.5 cm, Pharmacia) with a step gradient at 60 mM imidazole, and proteins were eluted from the column in buffer A with 500 mM imidazole. For StrepII-tagged proteins, cells were lysed and applied to a StrepTactin column (0.5 × 5 cm, Qiagen) in buffer A. Proteins were eluted from the column in buffer A with 2.5 mM desthiobiotin. For cells expressing both StrepII- and His_6_-tagged proteins, “two-step” purification was performed which incorporated sequential Ni-NTA and StrepTactin chromatography [38]. Native molecular mass of Rio1p was determined by applying protein fractions eluted from a StrepTactin column to a calibrated Superose 200 HR 10/30 column as recommended by supplier (Pharmacia-GE Healthcare, Piscataway, NJ). Molecular mass standards for calibration included cytochrome c (12.4 kDa), carbonic anhydrase (29 kDa), bovine serum albumin (66 kDa), alcohol dehydrogenase (150 kDa), *β*-amylase (200 kDa), and apoferritin (443 kDa) (Sigma). For molecular mass standards, the gel filtration column was equilibrated in 20 mM Tris buffer at pH 7.2 supplemented with 150 mM NaCl. Salt concentration was increased to 2 M NaCl for Rio1 kinase.

### 2.5. Two-Dimensional Gel Electrophoresis and Western Blot Analysis

TRIzol-extracted and benzonase-treated protein (up to 250 *μ*g) was applied to an 11 cm immobilized pH gradient (IPG) strip with a pH range of 3.9–5.1 (Bio-Rad). Proteins were separated according to their pI using manufacturer's protocol and previously published methods [39]. Proteins were further separated using either 10% or 12% SDS-PAGE Criterion gels (Bio-Rad) at 16°C at 200 V for 50 or 70 minutes, respectively. Proteins were transferred from gels to PVDF membranes at 100 V and 4°C for 90–100 minutes in 10 mM 2-(N-morpholino) ethanesulfonic acid (MES) buffer at pH 6.0 supplemented with 10% (vol/vol) methanol. Polyclonal antibodies raised in rabbit against PAN-A and *α*1 and phosphoamino monoclonal antibodies (clone 22a, Becton Dickinson Biosciences, San Jose, CA) were used for Western blot analysis as previously described [37].

### 2.6. Phosphatase Treatment

Protein (up to 80 *μ*g) was incubated (30°C, 20 minutes) in the presence and absence of lambda phosphatase (400 U) in 100 *μ*L 50 mM Tris buffer at pH 7.5 supplemented with 0.1 mM EDTA, 5 mM dithiothreitol and 2 mM MnCl_2_ (lambda phosphatase buffer).

### 2.7. Peptidase Assay

Chymotrypsin-like peptide hydrolyzing activity was assayed by release of 7-amino-4-methylcoumarin from N-Suc-Leu-Leu-Val-Tyr-7-amido-4-methylcoumarin (Suc-LLVY-AMC; Sigma) at 60°C for 10–30 minutes by an increase in fluorescence (excitation = 348 nm, emission = 440 nm) as previously described [40]. The assay mixture (0.3 mL) contained 1–3 *μ*g protein and 20 *μ*M fluorogenic substrate in buffer A with 0.4% (v/v) dimethyl sulfoxide (DMSO).

### 2.8. Kinase Assay

A Rio1p kinase homolog (HVO_0135) was expressed in *H. volcanii* with a C-terminal StrepII tag (-WSHPQFEK) that included a GT linker (encoded by a KpnI site) and purified to electrophoretic homogeneity by StrepTactin chromatography as described above. The purified kinase was dialyzed into 20 mM Tris buffer (pH 7.2) with 2 M NaCl or 2 M KCl at 4°C using a D-tube dialysis tube (EMD Chemicals, Gibbstown, NJ). Autophosphorylation was monitored by incubating purified Rio1p (1 *μ*g) with MnCl_2_ or MgCl_2_ (0–250 mM) for one hour at 37°C in the presence of 200–400 *μ*Ci of [*γ*-^32^P] adenosine 5′-triphosphate (ATP) (Perkin Elmer) in a reaction volume of 20 *μ*L. For *in vitro* phosphorylation, 10 *μ*g of *α*1 variants were included in the assay mixture. Kinase reactions were inactived by addition of an equal volume of reducing SDS-PAGE loading buffer and separated by 12.5% SDS-PAGE gels. Gels were stained with Coomassie brilliant blue and dried to Whatman paper. Blue sensitive X-ray film (Research Products International Corp., Mt. Prospect, IL) was exposed to the dried gel for 1–10 days at −80°C.

### 2.9. Mass Spectrometry

Proteasomal fractions (3 to 6 *μ*g protein) from nickel affinity chromatography and/or two-step affinity chromatography were separated by reducing 12% SDS-PAGE. Gels were stained with Bio-Safe Coomassie (Bio-Rad). Protein bands corresponding to the molecular masses of the CP subunits were excised from the gel and destained with 100 mM NH_4_HCO_3_ in 50% (vol/vol) acetonitrile (4°C, overnight). Protein samples were reduced, alkylated in-gel, and digested with trypsin (Promega, Madison, WI). Capillary reverse-phase high-performance liquid chromatography (HPLC) separation of the protein digests was performed using a PepMap C18 column (75-*μ*m inside diameter, 15-cm length; LC Packings, San Francisco, CA) with an Ultimate capillary HPLC system (LC Packings). A gradient (90 or 120 minutes) from 5 to 50% acetonitrile in 0.1% acetic acid was used at a flow rate of 200 nL per min. Tandem mass spectrometry (MS/MS) analysis was performed using a hybrid quadrupole time-of-flight (QTOF) instrument (QSTAR) equipped with a nanoelectrospray source and operated with Analyst QS 1.1 data acquisition software (Applied Biosystems).

### 2.10. Database Searching

Tandem mass spectrometry (MS/MS) data generated by information-dependent acquisition with QSTAR were searched against the deduced proteome of the *H. volcanii *DS2 genome sequence [41] by using the Mascot (Matrix Science, Boston, MA) database search engine. Probability-based Mascot scores were determined by a comparison of search results against estimated random match population and are reported as ~10 ×log _10_ (*p*), where *p* is the absolute probability. Individual Mascot ion scores greater than 32 were considered to indicate identity or extensive homology (*P* < .05). Scores below this default significant value were considered for protein identification in addition to validation by manual interpretation of MS/MS spectra. Carbamidomethylation of cysteine was used as a fixed modification based on sample preparation. Variable modifications that were implemented in the database search included deamidation of asparagine and glutamine, N-terminal acetylation, oxidation (single and double) of methionine, and phosphorylation of serine, threonine, and tyrosine.

## 3. Results

### 3.1. Isoforms of *α*1 Modulated during Growth-Phase Transitions

**Figure 1:**
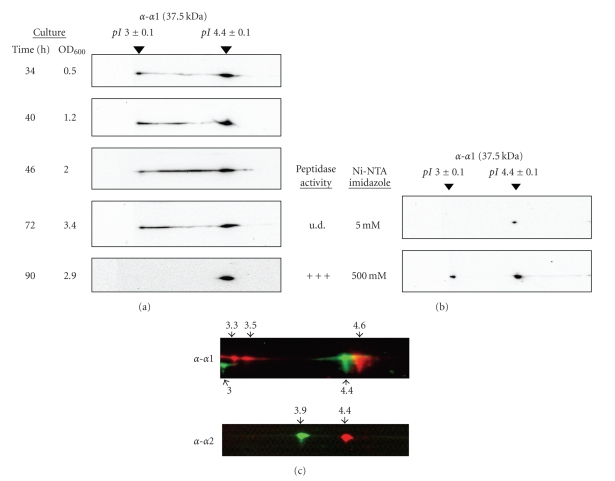
Phosphatase-sensitive isoforms of *α*-type proteins of proteasomal CPs as a function of growth in *H. volcanii* cells. (a) An *α*1 isoform of pI 4.4 was present at all stages of growth with a more acidic isoform of pI 3.0 present in log- and early-stationary phase, but absent in late-stationary phase. Cell lyase was prepared from various stages of growth as indicated (1 OD_600_ unit ~1 × 10^9^ CFU·mL^−1^), separated by 2-DE and analyzed by immunoblot using anti-*α*1 antibody (*α*-*α*1). (b) The two *α*1 isoforms of pI 3.0 and 4.4 are associated in proteasomal CPs. Proteasomal CPs were purified by Ni-NTA chromatography from early stationary-phase cells expressing *β*-His_6_. Proteins were separated into two fractions: (i) flowed through the Ni-NTA column at 5 mM imidazole and (ii) bound and were eluted from the column at 500 mM imidazole. Protein fractions were separated by 2-DE and analyzed by immunoblot using anti-*α*1 antibody (*α*-*α*1). Fractions were also assayed for peptidase activity using Suc-LLVY-AMC. u.d., undetectable. (c) Both *α*1 and *α*2 isoforms are sensitive to phosphatase treatment. Proteasomal CPs (purified as above) were treated with (red) and without (green) phosphatase, separated by 2-DE, and probed by immunoblot with anti-*α*1 and anti-*α*2 antibodies as indicated (*α*-*α*1 and *α*-*α*2, resp.).

Posttranslational modification of *α*1, one of the predominant proteasomal proteins in *H. volcanii *cells, was examined during various stages of growth from log- to late-stationary phase by 2-DE immunoblot of cell lysate. Two *α*1-specific isoforms of pI 3.0 and 4.4 were detected, both of which migrated at 37.5 kDa consistent with the mobility of the *α*1 protein in reducing SDS-PAGE gels [34]. The *α*1-isoform of pI 4.4 was present at all stages of growth while the second more acidic *α*1-isoform of pI 3.0 was present in earlier phases of growth and disappeared in late-stationary phase (Figure 1(a)).

Affinity purification of CPs from early stationary-phase cells expressing a hexahistidine tagged (-His_6_) derivative of the catalytic *β* subunit revealed that both isoforms of *α*1 (pI 3.0 and 4.4) associate as CPs active in hydrolysis of the peptide reporter Suc-LLVY-AMC (Figure 1(b)). The *α*1 isoform of pI 4.4 was also detected in non-CP fractions. Treatment of these purified 20S CPs with phosphatase resulted in a basic shift in pI of both *α*1 and *α*2 proteins signifying both *α*-type subunits were phosphorylated (Figure 1(c)).

Together these results suggest that the *α*-type proteins of archaeal proteasomal CPs can be modified by phosphorylation. Our previous studies demonstrate that the *β* subunits of *H. volcanii* CPs are also phosphorylated at Ser129 [35]. Thus, phosphorylation appears to be a major modification of the proteasome system of this haloarchaeon. Based on the isoelectric focusing (IEF) migration and phosphatase sensitivity of both *α*1 isoforms of pI 3.0 and 4.4, we propose that *α*1 harbors at least two phosphosites. The least phosphorylated form of *α*1 (pI 4.4) was detected at all stages of growth and occurred in both free and CP-associated forms. In contrast, the more extensively phosphorylated form of *α*1 (pI 3.0) was only detected in association with CPs and was not detected in late-stationary phase. In addition to these modified forms of *α*1, CP subtypes appear to incorporate a phosphorylated form of *α* *2*. Thus, *H. volcanii* not only modulates the types of subunits present in the CP and PAN subtypes during the transition from log- to stationary-phase growth [37], but appears also to posttranslationally modify the CP proteasomal proteins during this transition.

### 3.2. *α*1, *α*2, and PAN-A Are Phosphorylated at Ser/Thr Residues

To further map the phosphosites of CPs and PAN complexes, the various subunits (*α*1, *α*2, *β*, PAN-A, and PAN-B) were enriched from *H. volcanii* by nickel chromatography using His_6_ epitope-tags and analyzed by MS after tryptic digest. Initially, CPs were purified from log-phase *H. volcanii* H26-pJAM204 cells expressing *α*1-His_6_ and separated by SDS-PAGE prior to RP-HPLC MS/MS-analysis. Using this approach, a high-probability phosphopeptide was reproducibly detected in the *α*1-specific protein band that mapped to amino acid residues 137 to 149 of *α*1 (ALLIGGVENGpTPR, where pT represents phospho-Thr147) (Supplementary Figure 1). MS/MS-fragmentation of this peptide included a characteristic neutral loss (−98) indicative of CID removal of the phosphoryl group and confirmed that Thr147 was a site of *α*1 phosphorylation. The Thr147 residue was conserved in eukaryotic *α*-type CP proteins and was predicted to be phosphorylated by NetPhos (with a score of 0.851). An *α*2-specific phosphopeptide was also detected by precursor ion scanning of *α*2-specific tryptic peptides purified from *H. volcanii* H26-pJAM205 cells expressing *α*2-His_6_. The *α*2-specific phosphopeptide was composed of amino acid residues 12 to 21 (GTSLFSPDGR) and again displayed a neutral loss characteristic of phosphorylated peptides (Supplementary Figure 2). MS/MS, however, could not distinguish between Thr13 and Ser14 as the phosphosite due to the close vicinity of these residues within the peptide. Of these two residues, Ser14 has a higher probability of serving as a phosphosite compared to Thr13 based on modeling to known eukaryotic phosphoproteins with NetPhos scores of 0.957 and 0.243, respectively. In addition to the *α*1- and *α*2-specific phosphopeptides, a PAN-A-specific phosphopeptide was reproducibly detected by ESI-QTOF analysis of tryptic digestions of PAN complexes purified from *H. volcanii* expressing either PAN-A-His_6_ or PAN-B-His_6_ (DS70-pJAM650 or pJAM1012, resp.) (Supplementary Figure 3). This phosphopeptide mapped to amino acid residues 337 to 361 of PAN-A (MNVpSDDVDFVELAEMADNASGADIK, where pS represents phospho-Ser340). MS/MS fragmentation confirmed that Ser340 was the phosphorylated amino acid including a neutral loss peak (−98 for phosphorylation) and several internal fragmentation ions. It is important to note that the PAN-A-Ser340-specific phosphopeptide was detected in strains expressing either PAN-A-His_6_ or PAN-B-His_6_. This indicates that phosphorylated PAN-A associates with PAN-B in *H. volcanii* cells. Interestingly, the PAN-A Ser340 and amino acids surrounding this residue are highly conserved among eukaryotic 26S proteasome Rpt-like proteins including those of humans; however, the prediction of this as a phoshosite by NetPhos was low with a score of 0.464. Similar to the Ser129 phosphosite that was mapped to the CP *β* subunit [35], analogous nonphosphorylated peptides also mapped to *α*1 Thr147, *α*2 Thr13/Ser14, and PAN-A Ser340 residues. These results suggest that the phosphosites of PAN and CP complexes are not fully occupied by phosphoryl groups and, thus, the phosphorylation of proteasomes may be regulated in the cell.

### 3.3. Variant *α*1 Proteins Affect Pigmentation and Cell Viability

Single and double knock-out of *psmA* (*α*1) and *panA* (PAN-A) genes diminishes the ability of *H. volcanii* cells to adapt to low salt and other stressful conditions [5]. These phenotypes are readily complemented by providing a copy of the corresponding wild type gene in *trans* and, thus, provide an ideal assay for examining how phosphorylation may influence in the function of *α*1 and PAN-A in the cell.

Initially, *panA* mutant cells were tested for their ability to be complemented for growth on low-salt media with *panB*, *panA,* or *pan* *A*_*t*1018*g*_ (PAN-A Ser340Ala) genes. For complementation, the genes were expressed in *trans* from a multicopy pHV2-based plasmid using a strong rRNA P2 promoter. While overexpression of *panB* from the plasmid did not rescue the sensitivity of the *panA* mutant to low salt conditions, expression of either *panA* or *panA *Ser340Ala restored growth to wild-type levels. This indicates that phosphorylation of Ser340 is not important for response to hypo-osmotic stress (data not shown) and suggests that *panB* cannot substitute for *panA* under these growth conditions.

**Figure 2:**
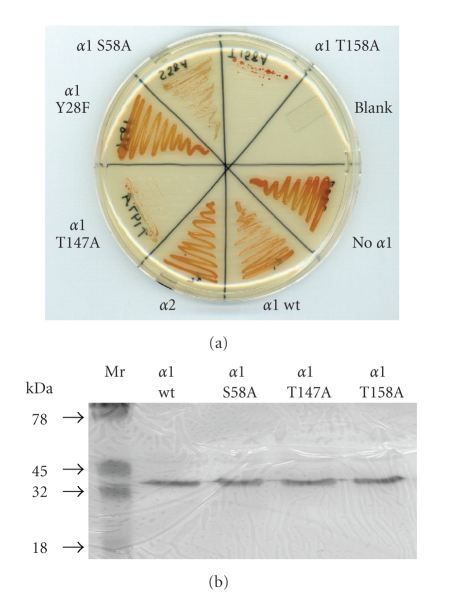
Analysis of an *H. volcanii psmA* (*α*1) knockout expressing wild type and variant *α*-type proteasomal genes *in trans*. (a) Growth and pigmentation of an *H. volcanii psmA *mutant (GZ130) were influenced by expression of *α*1 variant proteins on ATCC novobiocin plates. Synthesis of *α*1 Thr147Ala and *α*1 Thr158Ala inhibited growth of the *psmA* mutant, while *α*1 Ser58Ala and *α*1 Thr147Ala altered pigmentation. (b) No significant difference in *α*1 protein levels were detected in the *H. volcanii psmA* mutant synthesizing the wild type and variant *α*1 proteins from genes provided *in trans*. The *α*-type proteins encoded by the genes expressed *in trans* are indicated with the following abbreviations: wt, wild type; S58A, Ser58Ala; Y28F, Tyr28Phe; T158A, Thr158Ala; T147A, Thr147Ala.

Site-directed mutations in the *psmA* gene encoding single amino acid substitutions of Ser58Ala, Thr147Ala, Thr158Ala, and Tyr28Phe in *α*1 were tested (along with overexpression of *psmC* encoding *α*2) for their ability to complement the hypo-osmotic stress response phenotype of a *psmA* mutant. Similar to *panA*, the modified *psmA* and *psmC* genes were provided in *trans* using a pHV2-based plasmid and strong rRNA P2 promoter. The *α*1-residues selected for site-directed modification were based on the following: *α*1 Thr147 was detected in this study as a phosphosite by MS/MS (see above for details), *α*1 Tyr28 was homologous to the Tyr27 phosphosite identified in the *α*2-type subunit of eukaryotic CPs [22], and Ser58 and Thr158 are predicted to be phosphorylated based on on NetPhos 2.0 scores of 0.996 and 0.981, respectively, (significantly above the significance threshold of 0.500) [42]. Overexpression of the *psmC* (*α*2) gene did not complement the *psmA* mutant while the *psmA* and *psmA-a83t* (*α*1 Tyr28Phe) genes complemented the reduced growth phenotype of the *psmA *mutant on low salt media to wild type levels. In contrast, the other substitutions to the *psmA* gene provided *in trans* severely inhibited overall growth of the *psmA* mutant strain. While each of the constructs was successfully transformed into the *psmA* knockout strain, growth of the transformed cells was drastically reduced after being transferred to liquid media or streaked onto a fresh plate (Figure 2(a)). Some of the substitutions in *α*1 including Ser58Ala and *α*1 Thr147Ala appeared to affect carotenoid biosynthesis as cells expressing these *α*1 variants were whiter than cells expressing wild-type *α*1 or no *α*1 at all (Figure 2(a)). Whole cell chymotrypsin-like (CL) peptidase activity of the different *α*1 substitution mutants expressed *in trans* was compared to wild type after initial transfer to liquid media. Only modest differences in specific activity were detected between the strains with the most pronounced difference in cells expressing *α*1 Thr158Ala, which had a ~2-fold increase in CL activity compared to wild-type (data not shown). It is unclear at this time if these modest differences in CL activity can account for the phenotypes observed. Anti-*α*1 immunoblot of cell lysate revealed all of the *α*1 variant proteins were produced at levels similar to wild type when the *psmA* genes were expressed *in trans* in the *psmA *knockout (Figure 2(b)). Both the hypo-osmotic stress and pigmentation phenotypes were recessive based on the observation that these *α*1 variants did not alter growth or pigmentation when expressed *in trans* in wild-type *H. volcanii* cells (data not shown).

### 3.4. *Haloferax volcanii* Encodes a Number of Putative Protein Kinases

**Figure 3:**
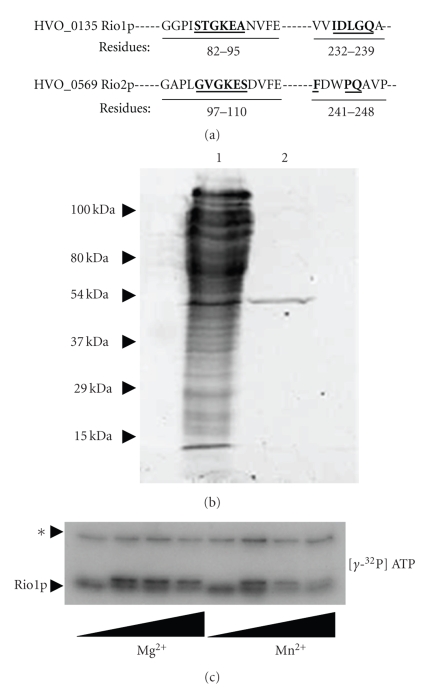
Rio-type protein kinases of *H. volcanii*. (a) Rio-type protein kinase homologs of *H. volcanii*, type-1 (HVO_0135) and type-2-like (HVO_0569), with conserved amino acid residues in bold and underlined. While HVO_0569 does not have a complete C-terminal domain, it does contain three of the five conserved residues of Rio type-2 kinases. (b) Purification of Rio1p (HVO_0135) from *H. volcanii*. Reducing SDS-PAGE gel of cell lysate (lane 1) and Rio1p purified by StrepTactin chromatography (lane 2) from in *H. volcanii*-pJAM2558. (c) Rio1p catalyzes its autophosphorylation. Autophosphotransfer was detected *in vitro* by autoradiography using [*γ*-^32^P] ATP as a substrate with optimal activity detected at 10 to 50 mM MgCl_2_. *, identity of band remains to be determined.

Based on genome sequence, *H. volcanii* encodes for a number of putative serine/threonine kinases including two different Rio-type protein kinases, type-1 (HVO_0135) and type-2-like (HVO_0569) (Figure 3(a)) as well as homologs of the serine protein kinase PrkA, originally described in *Bacillus subtilis* [43]. Rio-type kinases are a relatively new atypical protein kinase family with four unique types, Rio1, Rio2, Rio3, and RioB. Rio-type kinases are widespread throughout eukaryotes and archaea. Archaea typically have two different Rio-type kinases, Rio1 and Rio2 [44]. Rio1 kinases are characterized by a consensus sequence STGKEA in the N-terminal domain of the protein and a second region of homology in the C-terminal domain, IDxxQ (where x represents any amino acid residue). While the Rio2 kinases can vary somewhat in domain structure, these kinases are characterized by a helix-turn-helix motif in the N-terminus followed by the amino acid sequence GxGKES and a C-terminal IDFPQ sequence [45]. The Rio1p from *H. volcanii* contains the signature domain sequence but the Rio2p does not (Figure 3(a)). 

### 3.5. *H. volcanii* Rio Type-I Kinase Catalyzes Autophosphorylation

To investigate the activity of the Rio1p of *H. volcanii*, the C-terminal residue of this kinase was fused to a StrepII tag and synthesized in recombinant *H. volcanii* (H26-pJAM2558). Rio1p was purified to electrophoretic homogeneity by StrepTactin chromatography (Figure 3(b)) and determined to be a 50-kDa monomer based on gel filtration chromatography compared to migration by reducing SDS-PAGE and deduced polypeptide sequence. Rio1p catalyzed its autophosphorylation in the presence of either Mg^2+^ or Mn^2+^ as the divalent cation (Figure 3(c)). The ideal concentration of cation was 10 to 50 mM Mg^2+^ in 20 mM Tris-buffer pH 7.2 supplemented with 2 M NaCl with 4 *μ*M enzyme. The levels of Rio1p autokinase activity were comparable when assayed in the presence of either 2 M KCl or NaCl (data not shown). All subsequent kinase reactions were carried out in 20 mM Tris buffer (pH 7.2) with 2 M NaCl and 50 mM MgCl_2_. Multiple bands were visible on autoradiographs after the Rio1p-mediated autophosphorylation reaction including a doublet at the apparent molecular mass of the Rio1p monomer in addition to a band running at approximately twice the mass. MS analysis of Rio1p (from solution) did not identify any contaminating proteins in the purified fraction (Supplementary Table 3). The multiple phospho-bands observed after assay may represent a transient dimer that forms between two Rio1p proteins in the presence of ATP. It is also possible that there is a contaminating protein that is phosphorylated in the reaction mixture and is not detected by MS or SYPRO staining.

### 3.6. Rio1p Phosphorylates *α*1 Proteins *In Vitro*

**Figure 4:**
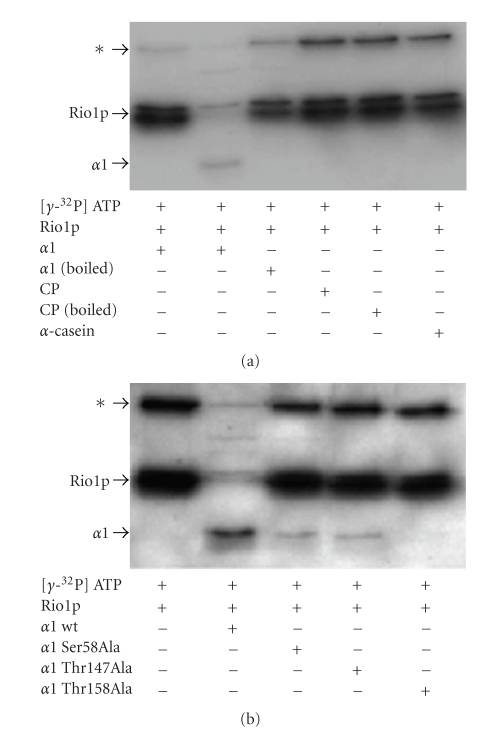
Rio1p phosphorylates *α*1 at Ser and Thr residues. (a) Phosphotransfer of Rio1p to *α*1 is catalyzed using *α*1-His_6_ from recombinant *E. coli* as a substrate. Phosphotransfer was assayed using the protein substrates indicated as follows: *α*1-His_6_ (boiled and unboiled) from recombinant *E. coli*, 20S CPs of *α*1-His_6_ and *β*-StrepII subunits (boiled and unboiled) from *H. volcanii* and *α*-casein from bovine milk (boiled), as indicated. (b) Phosphotransfer of Rio1p to *α*1 is reduced by site-directed *α*1 protein variants Ser58Ala, Thr147Ala and Thr158Ala. The *α*1 wild type and protein variants were purified from recombinant *E. coli* and were not boiled prior to assay. Phosphotransfer was detected by autoradiography using Rio1p-StrepII purified by StrepTactin chromatography and *γ*-^32^P P-ATP as a substrate.

Purified Rio1p was assayed for its ability to phosphorylate *α*1-His_6_ purified from recombinant *E. coli*, 20S CPs purified from *H. volcanii* and *α*-casein from bovine milk. While the *α*1-proteins purified from *E. coli* (*α*1_EC_) form a mixture of hexameric rings and monomers, these recombinant proteins are not anticipated to be phosphorylated in recombinant *E. coli* based on their migration as a single spot by 2-DE (data not shown) as well as the distant phylogenetic relationship between *E. coli* and *H. volcanii*. Rio1p phosphorylated the unboiled *α*1_EC_ preparation but was unable to phosphorylate the CPs or boiled *α*1_EC_ or *α*-casein (Figure 4(a)). When equal amounts of *α*1_EC_ and site-directed *α*1 variants all purified from *E. coli* were used in the kinase assay, varying levels of *α*1-phosphorylation were observed. Wild-type *α*1_EC_ was the most heavily phosphorylated under these conditions. Both *α*1_EC_ Ser58Ala and *α*1_EC_ Thr147Ala were phosphorylated but to a lesser extent than wild-type *α*1_EC_ (2.5-fold lower based on densitometry of autoradiograph). The variant *α*1_EC_ Thr158Ala was not phosphorylated under these conditions (Figure 4(b)). Based on these results, coupled with our earlier findings by 2-DE, *α*1 is most likely phosphorylated at 2 or 3 different sites. It is unclear at this time why *α*1_EC_ Thr158Ala was not phoshporylated by Rio1p *in vitro*. There may be drastic structural changes in this *α*1 protein that prevent meaningful interaction with the kinase.

### 3.7. *α*1 Is Methylesterified on Acidic Residues

In addition to phosphorylation, the *α*1 protein was also methylated. Five unique methylation sites were reproducibly detected with high probability by MS/MS for *α*1 (Asp20, Glu27, Glu62, Glu112, and Glu161) (Supplementary Figure 4). Substantiating this finding, several of the MS/MS-fragmentations also showed neutral losses of methyl groups (−14). Unmodified *α*1 peptides that mapped to the same residues as those that were methylated were also detected. The analogous peptide in its unmodified state was almost always present in the same preparation, suggesting that methylated and unmethylated *α*1 proteins exist simultaneously in the cell. O-linked methylesterification is a reversible posttranslational modification, and therefore *α*1 methylation may be regulated. However, there was no clear correlation to growth phase or environmental conditions examined that increased or decreased the frequency of the methylated state of *α*1 protein (data not shown).

The *psmA* gene, encoding for the CP *α*1 protein, is cotranscribed with a putative S-adenylosylmethionine-dependent methyltransferase (HVO_1093) [46]. Whether this enzyme mediates *α*1 methylation remains to be determined; its *in vitro* *α*1-metylating activity could not be demonstrated (data not shown). Future work is needed to better understand how and why this modification takes place.

## 4. Discussion

Our previous work established that the three CP subunits of *H. volcanii* proteasomes (*α*1, *α*2, and *β*) are modified post- and/or co-translational [34, 35]. The *β* subunit is phosphorylated and cleaved to expose the active site Thr, and the *α*1 and *α*2 subunits are N^*α*^-acetylated on the initiator methionine residue. However, additional modifications were suspected based on the detection of long isoform chains of CP proteins including at least four *α*1 isoforms that were separated by 2-DE [35].

In this study, the covalent modification of archaeal proteasomal proteins was further examined using *H. volcanii* as a model system. Additional sites of posttranslational modification were identified including the phosphorylation of *α*1, *α*2, and PAN-A as well as the methyl esterification of *α*1. The phosphorylation of *α*1 was temporally regulated with the number of phosphatase-sensitive *α*1 isoforms reduced during late-stationary phase. The most acidic, presumably most phosphorylated isoform of *α*1, was only detected in association with *β* as 20S CPs and was not detected in late-stationary phase.

In addition to elaborating on the prevalence and temporal nature of archaeal proteasomal PTMs, a member of the Rio-kinase family, Rio1p, was demonstrated to catalyze the phosphorylation of *α*1 *in vitro*. Modification of Ser58, Thr147, or Thr158 residues of *α*1 to alanine significantly reduced this Rio1p-dependent phosphotransfer activity. Although the finding that Rio1p phosphorylates *α*1 *in vitro* does not imply that this interaction is physiological, preliminary data on this substrate/kinase pair may prove valuable in understanding the role phosphorylation plays in regulating 20S CPs or other proteins in archaea. Substrate/kinase pairs are rarely known in this domain of life [47].

In vivo expression of phosphosite mutant forms of *α*1 revealed a biological connection between the phosphorylation of proteasomes and the pigmentation and viability of archaeal cells. While the details of this link remain to be determined at the molecular level, cell division is known to be highly regulated by the ubiquitin (Ub)-proteasome system in eukaryotic cells [48]. In addition, 3-hydroxy-3-methylglutaryl coenzyme A (HMG-CoA) reductase, a key enzyme in the mevalonate pathway for production of isoprenoids (including carotenoids) is heavily regulated by proteasomes [49], and the Ub ligase CrgA is required for proper regulation of carotenogenesis in fungi [50].

In addition to phosphorylation, the *α*1 subunit of 20S CPs was found to be heavily methylated in *H. volcanii*. Although methylation has not been previously reported as a PTM of proteasomes in any organism, there are examples of reversible methylation of proteins in bacteria and archaea. Like bacteria, the haloarchaea have several taxis proteins that are O-methylated resulting in altered protein-protein interactions that enable cells to respond to external stimuli [51–53]. Haloarchaea may regulate the activity of other systems, such as proteasomes, using a similar strategy. Alternatively, the methyl esterification of *α*1 may be used to reversibly modulate the overall charge of proteasomal CPs. Most haloarchaeal proteins, including *α*1, are highly acidic (low pI) with the acidic residues typically on the surface of the protein forming a hydration shell. This shell aids in “salting-in” the protein and maintaining the activity of the protein at the unusually high concentrations of salt found in the cytosol of these microorganisms. Although we did not detect quantitative differences in the methylation state of *α*1 under different growth conditions, *α*1 is required for *H. volcanii* to properly respond to low salt stress [5]. Whether the methyl esterification of *α*1 modifies protein-protein interactions in regulatory mechanisms such as PAN : CP interactions, buffers *α*1 in the cytosol at different concentrations of salt and/or mediates other physiological processes remains to be determined.

## Supplementary Material

Supplementary material includes a list of the strains and plasmids used in this study, oligonucleotide primer pairs used for site-directed mutagenesis, MS/MS-results that support the homogeneity of Rio1p kinase from *H. volcanii*, and mass spectra used to identify the sites of post-translational modification of *H. volcanii* proteasomes including the phosphosites *α* 1 Thr147, *α* 2 Thr13/Ser14 and PAN-A Ser340 as well as the methylesterification sites *α* 1 Asp20, Glu27, Glu62, Glu112 and Glu161.
