# Supplementary material for: Phosphorylation and Methylation of Proteasomal Proteins of the Haloarcheon Haloferax volcanii
Source: Archaea. 2010 Jul 8;2010:481725. doi: 10.1155/2010/481725 (PMC2910475; doi:10.1155/2010/481725)

Suppl. Table 1. Strains and Plasmids used in this study.

Strain or plasmid	Phenotype, genotype, description, and/or PCR Primers[1]	Source or reference(s)[2]
**Strains:**		
E. coli		
DH5α	*fhuA2* Δ*(argF-lacZ)U169 phoA glnV44 Φ80* Δ*(lacZ)M15 gyrA96 recA1 relA1 endA1 thi-1 hsdR17*	Life Technologies
GM2163	*dam-13::*Tn *9 dcm-6 hsdR2 leuB6 his-4 thi-1 ara-14 lacY1 galK2 galT22 xyl-5 mtl-1 rpsL136 tonA31 tsx-78 supE44* $McrA^-$ $McrB^-$	New England Biolabs
BL21 (DE3)	F^- *ompT* [*lon*] *hsd* S_B ($r_B^- m_B^-$) (an *E. coli* B strain) with DE3, a λ prophage carrying the T7 RNA polymerase gene	Novagen
H. volcanii		
H26	DS70 Δ*pyrE2*	[1]
GZ109	H26 Δ*panA*	[2]
GZ114	H26 Δ*psmC*	[2]
GZ130	H26 Δ*psmA*	[2]
GZ138	H26 P_{tnaA}-*psmB*	[2]
**Plasmids:**		
pET24b	Km^r; *Eco* expression vector	Novagen
pJAM202	Ap^r; Nv^r; pBAP5010 $P2_{rrn}$-*psmB-his*$_6$ (β-His_6) expressed in *Hvo*	[3]
pJAM204	Ap^r; Nv^r; pBAP5010 $P2_{rrn}$-*psmA-his*$_6$ (α1-His_6) expressed in *Hvo*	[3]
pJAM205	Ap^r; Nv^r; pBAP5010 $P2_{rrn}$-*psmC-his*$_6$ (α2-His_6) expressed in *Hvo*	[3]
pJAM621	Km^r; pET24b *psmB-his6* (β-His_6) expressed in *Eco*	[3]
pJAM622	Km^r; pET24b *psmA-his6* (α1-His_6) expressed in *Eco*	[3]
pJAM623	Km^r; pET24b *psmC-his6* (α2-His_6) expressed in *Eco*	[3]
pJAM648	Ap^r; Nv^r; pBAP5010 $P2_{rrn}$-*panA* (PanA) expressed in *Hvo*	[2]
pJAM650	Ap^r; Nv^r; pBAP5010 $P2_{rrn}$-*panA-his*$_6$ (PanA-His_6) expressed in *Hvo*	This study
pJAM816	Ap^r; Nv^r; pJAM809 $P2_{rrn}$-*psmB-strepII* (β-StrepII) expressed in *Hvo*	[4]
pJAM1012	Ap^r; Nv^r; pBAP5010 $P2_{rrn}$-*panB-his*$_6$ (PanB-His_6) expressed in *Hvo*	This study
pJAM2521	Km^r; pET24b *psmA-t172g-his6* (α1-S58A-His_6) expressed in *Eco*	This study
pJAM2522	Ap^r; Nv^r; pBAP5010 $P2_{rrn}$-*psmA-t172g-his6* (α1-S58A-His_6) expressed in *Hvo*	This study
pJAM2523	Km^r; pET24b *psmA-a471g-his6* (α1-T158A-His_6) expressed in *Eco*	This study
pJAM2524	Ap^r; Nv^r; pBAP5010 $P2_{rrn}$- *psmA-a471g-his6* (α1-T158A-His_6) expressed in *Hvo*	This study
pJAM2525	Km^r; pET24b *psmC-t40g-his6* (α2-S14A-His_6) expressed in *Eco*	This study
pJAM2526	Ap^r; Nv^r; pBAP5010 $P2_{rrn}$-*psmC-t40g-his6* (α2-S14A-His_6) expressed in *Hvo*	This study
pJAM2527	Km^r; pET24b *psmC-a235g-his6* (α2-T79A-His_6) expressed in *Eco*	This study

pJAM2528	Ap[r]; Nv[r]; pBAP5010 P2$_{rrn}$-*psmC-a235g-his6* (α2-T79A-His$_6$) expressed in *Hvo*	This study
pJAM2529	Km[r]; pET24b *psmB-t385g-his6* (β-S129A-His$_6$) expressed in *Eco*	This study
pJAM2530	Ap[r]; Nv[r]; pBAP5010 P2$_{rrn}$-*psmB-t385g-his6* (β-S129A-His$_6$) expressed in *Hvo*	This study
pJAM2531	Km[r]; pET24b *psmB-a388g-his6* (β- T130A-His$_6$) expressed in *Eco*	This study
pJAM2532	Ap[r]; Nv[r]; pBAP5010 P2$_{rrn}$- *psmB-a388g-his6* (β-T130A-His$_6$) expressed in *Hvo*	This study
pJAM2533	Km[r]; pET24b *psmA-a83t-his6* (α1-Y28F-His$_6$) expressed in *Eco*	This study
pJAM2534	Ap[r]; Nv[r]; pBAP5010 P2$_{rrn}$-*psmA-a83t-his6* (α1-Y28F-His$_6$) expressed in *Hvo*	This study
pJAM2537	Km[r]; pET24b *psmC-a37g-his6* (α2-T13A-His$_6$) expressed in *Eco*	This study
pJAM2538	Ap[r]; Nv[r]; pBAP5010 P2$_{rrn}$-*psmC-a37g-his6* (α2-T13A-His$_6$) expressed in *Hvo*	This study
pJAM2545	Ap[r]; Nv[r]; pJAM816 *psmB-strepII psmA-his$_6$* (β-StrepII, α1-His6) expressed in *Hvo*	[4]
pJAM2553	Ap[r]; Nv[r]; pBAP5010 P2$_{rrn}$-*panA-t1018g* (PanA-S340A) expressed in *Hvo*	This study
pJAM2554	Km[r]; pET24b *psmA-a439g-his6* (α1-T147A-His$_6$) expressed in *Eco*	This study
pJAM2555	Ap[r]; Nv[r]; pBAP5010 P2$_{rrn}$- *psmA-a439g-his6* (α1-T147A-His$_6$) expressed in *Hvo*	This study
pJAM2558	Ap[r]; Nv[r]; pJAM816 P2$_{rrn}$-*rio1p-strepII* (Rio1-StrepII) expressed in *Hvo*	This study

[1]Eco, *E. coli*. Hvo, *H. volcanii*.

[2]References.

[1] Allers, T., H.P. Ngo, M. Mevarech and R.G. Lloyd. 2004. Development of additional selectable markers for the halophilic archaeon *Haloferax volcanii* based on the *leuB* and *trpA* genes. Appl. Environ. Microbiol. 70:943-953.

[2] Zhou, G., D. Kowalczyk, M.A. Humbard, S. Rohatgi and J.A. Maupin-Furlow. 2008. Proteasomal components required for cell growth and stress responses in the haloarchaeon *Haloferax volcanii*. J. Bacteriol. 190:8096-8105.

[3] Kaczowka, S.J. and J.A. Maupin-Furlow. 2003. Subunit topology of two 20S proteasomes from *Haloferax volcanii*. J. Bacteriol. 185:165-174.

[4] Humbard, M.A., G. Zhou and J.A. Maupin-Furlow. 2009. The N-terminal penultimate residue of 20S proteasome α1 influences its Nα-acetylation and protein levels as well as growth rate and stress responses of *Haloferax volcanii*. J. Bacteriol. 191:3794-3803.

Suppl. Table 2. Oligonucleotide primer pairs for site-directed mutagenesis.

Mutation	Primer Sequence (5' > 3')
psmA-t172g (α1 S58A)	5'-CGGACAAGCGCTCTCGCGCGCCGCTGATGGAAC-3'
	5'-GTTCCATCAGCGGCGCGCGAGAGCGCTTGTCCG-3'
psmA-a471g (α1 T158A)	5'-GACCGACCCCTCGGGCGCCCCCTACGAGTGGAAGGC-3'
	5'-GCCTTCCACTCGTAGGGGGCGCCCGAGGGGTCGGTC-3'
psmA-a439g (α1 T147A)	5'-GGCGGCGTCGAAAACGGTGCGCCGCGCCTCTACGAG-3'
	5'-CTCGTAGAGGCGCGGCGCACCGTTTTCGACGCCGCC-3'
psmA-a83t (α1 Y28F)	5'-CTCTATCAGGTCGAATTCGCTCGTGAGGCCGTC-3'
	5'-GACGGCCTCACGAGCGAATTCGACCTGATAGAG-3'
psmB-t385g (β S129A)	5'-AGCATGCAGGCGCTGGCGACGCTCGTCGGCAACTTC-3'
	5'-GAAGTTGCCGACGAGCGTCGCCAGCGCCTGCATGCT-3'
psmB-a388g (β T130A)	5'-ATGCAGGCGCTGTCGGCGCTCGTCGGCAACTTCCTC-3'
	5'-GAGGAAGTTGCCGACGAGCGCCGACAGCGCCTGCAT-3'
psmC-a37g (α2 T13A)	5'-GCCTACGACCGCGGAGCGTCGCTTTTCTCCC-3'
	5'-GGGAGAAAAGCGACGCTCCGCGGTCGTAGGC-3'
psmC-t40g (α2 S14A)	5'-GCCTACGACCGCGGAACGGCGCTTTTCTCCCCCGAC-3'
	5'-GTCGGGGGAGAAAAGCGCCGTTCCGCGGTCGTAGGC-3'
psmC-a235g (α2 T79A)	5'-ACGCGCTCGGCGCGGCCGCGGCCGGCCACGTCGCC-3'
	5'-GGCGACGTGGCCGGCCGCGGCCGCGCCGAGCGCGT-3'
panA-t1018g	5'-ACCCGGAAGATGAACGTCGCCGACGACGTGGACTTCGTC-3'
(PanA S340A)	5'-GACGAAGTCCACGTCGTCGGCGACGTTCATCTTCCGGGT-3'

Suppl. Table 3. MS/MS analysis of Rio1p kinase fractions purified by Strep-Tactin chromatography.

Protein	Residue no.	MASCOT	E value	Sequence
Rio1p	185 – 192	23	0.033	DYLEGDPR
	221 – 233	40	0.00072	AGVRVPKPIAVQR
	193 – 205	23	0.034	FENIGHDKGQVVR
	234 – 249	17	0.15	NVLVMELVGVVDDRAR

Supplemental Figure Legends

Suppl. Fig. 1. α1 is phosphorylated at Thr147. An α1-specific phosphopeptide was detected in purified 20S proteasomal CPs by ESI-Q-ToF with precursor ion scanning. The peptide had a Mascot score of 30 and an expect (E) of 0.001. It was a doubly charged ion (m/z 479.3) mapping to a semi-tryptic fragment of α1 spanning amino acid residues 137 to 149 (ALLIGGVENGpTPR). Only one amino acid, Thr147, can be phosphorylated in that peptide, and MS/MS-annotation confirmed that Thr147 was the site of phosphorylation. The MS/MS fragmentation contained 7 y-ions and 6 out of the 7 showed the characteristic neutral loss (–98) indicative of a phosphorylation. Several other fragments ions could be assigned to internal sequencing ions of the peptide. These peak assignments bolster the MS/MS assignment. The peptide, although generated by a tryptic digest of a 20S proteasome preparation, was a semi-tryptic fragment, an internal chymotryptic-like cut on the N-terminus of the peptide. An additional scan using an error-tolerant algorithm for this phosphorylation site hit on two additional peptides; LIGGVENGpTP + 22 Da and LIGGVENGpT + 155 Da. The Mascot score for these two peptides were 50 and 58 and the E-values were 0.0006 and 0.001, respectively. The unmodified form of the peptide was also detected in the same fraction.

Suppl. Fig. 2. α2 is phosphorylated at Thr13 or Ser14. An α2-specific phosphopeptide was detected in purified 20S proteasomal CPs by MS/MS. The phosphopeptide, which eluted from RP-HPLC at 37.9 min, was detected as a doubly charged ion (m/z 558.2) by ESI-Q-ToF. The peptide had a Mascot score of 35 and an expect value (E) of 1.4e-3. The peptide was composed of amino acid residues 12 – 21 (GTSLFSPDGR) of α2. Unfortunately, MS/MS could not distinguish between Thr13 and Ser14 as the phosphosite. Although the MS/MS-fragmentation contained 6 unique y-ions and 3 unique b-ions, the close proximity of the phosphosite to the N-terminus of the peptide made the assignment difficult. A precursor ion scan was performed using a QQQ instrument (ABI QTRAP 4000) set to detect the neutral loss of a phosphate group. Several of the ion b-ions on the MS/MS fragmentation displayed the characteristic neutral loss (–98) for a phosphate, but the differentiating ions, b2 vs. y8 were missing in the spectrum. Since this phosphopeptide was detected by a precursor ion scan, the unmodified peptide was not detected in the same run. However, subsequent experiments have detected the unmodified form of this peptide.

Suppl. Fig. 3. PAN-A is phosphorylated at Ser340. A PAN-A-specific phosphopeptide was reproducibly detected by ESI-QTOF analysis of tryptic digestions of PAN-A purified from *H. volcanii strains* . The phosphopeptide eluted from RP-HPLC at 69.05 min as a quadrupoly charged ion (m/z 684.8, 4+) and was composed of amino acid residues 337 to 361 of PAN-A (MNVpSDDVDFVELAEMADNASGADIK). MS/MS fragmentation confirmed that Ser340 was the phosphorylated amino acid. Despite the large, 25-amino acid peptide, a high Mascot score (70) and low expect value (1.4e-7) were obtained due to the assignment of 9 y-ions and 6 b-ions in the MS/MS fragmentation. Additional peaks were assigned that increased the confidence of this peptide including a neutral loss peak (–98 for phosphorylation) and several internal fragmentation ions. The unmodified peptide was detected in the same preparations.

Suppl. Fig. 4. α1 is methylesterified on acidic residues Asp 20 (A), Glu 27 (B), Glu62 (C), Glu 112 (D) and Glu161 (E). Five unique methylated peptides were identified in several ESI-QTOF experiments of tryptic digestions of 20S proteasomes purified from *H. volcanii* DS70 strain cells. Each methylated peptide was specific for the α1 protein. The peptides mapped to amino acid residue numbers 13 to 22 (GITIFSPD$_{methyl}$GR), 23 to 30 (LYQVE$_{methyl}$YAR), 58 to 68 (SPLME$_{methyl}$PTSVEK), 105 to 116 (YGEPIGIE$_{methyl}$TLTK) and 150 to 163 (LYETDPSGTPYE$_{methyl}$WK). All five peptides eluted from the RP-HPLC as doubly charged ions: 538.77 (m/z), 528.23 (m/z), 616.28 (m/z), 667.82 (m/z) and 567.23 (m/z), with Mascot scores above 33 (up to 66) and E values from 2.5e-5 to 0.032. The high Mascot scores and corresponding low E-values validate the assignment of the peptides and the methylation sites. Several of the MS/MS-fragmentations also showed neutral losses of methyl groups (–14). Specifically, peptides 23 – 30 and 105 – 116 had three neutral loss peaks and the 58 – 68 peptide had one neutral loss peak. The MS/MS fragmentation was complete enough in all 5 spectra to definitively assign the location of the methylation.

Suppl. Fig. 1. α1 is phosphorylated at Thr147.

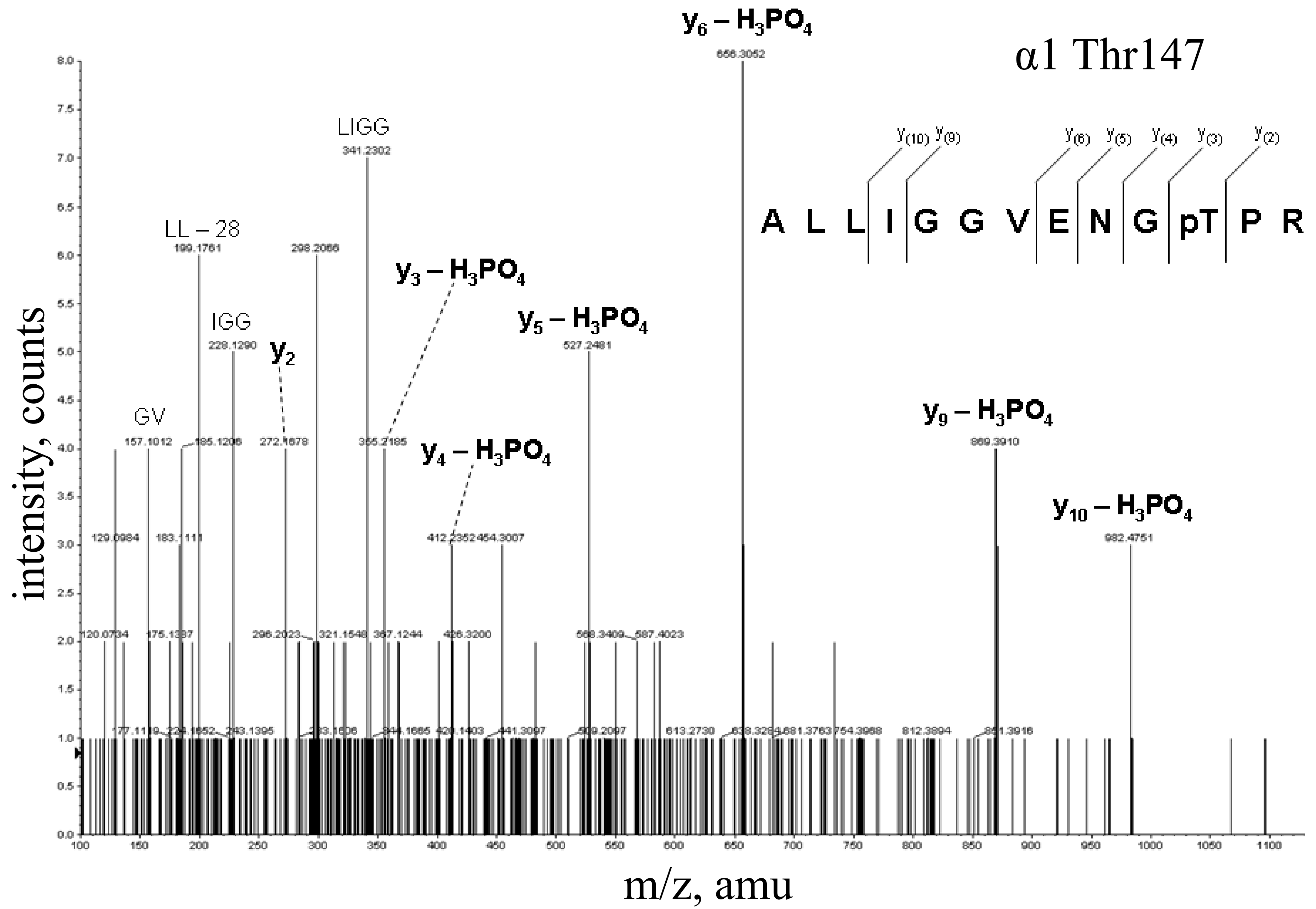

Suppl. Fig. 2. α2 is phosphorylated at Thr13 or Ser14.

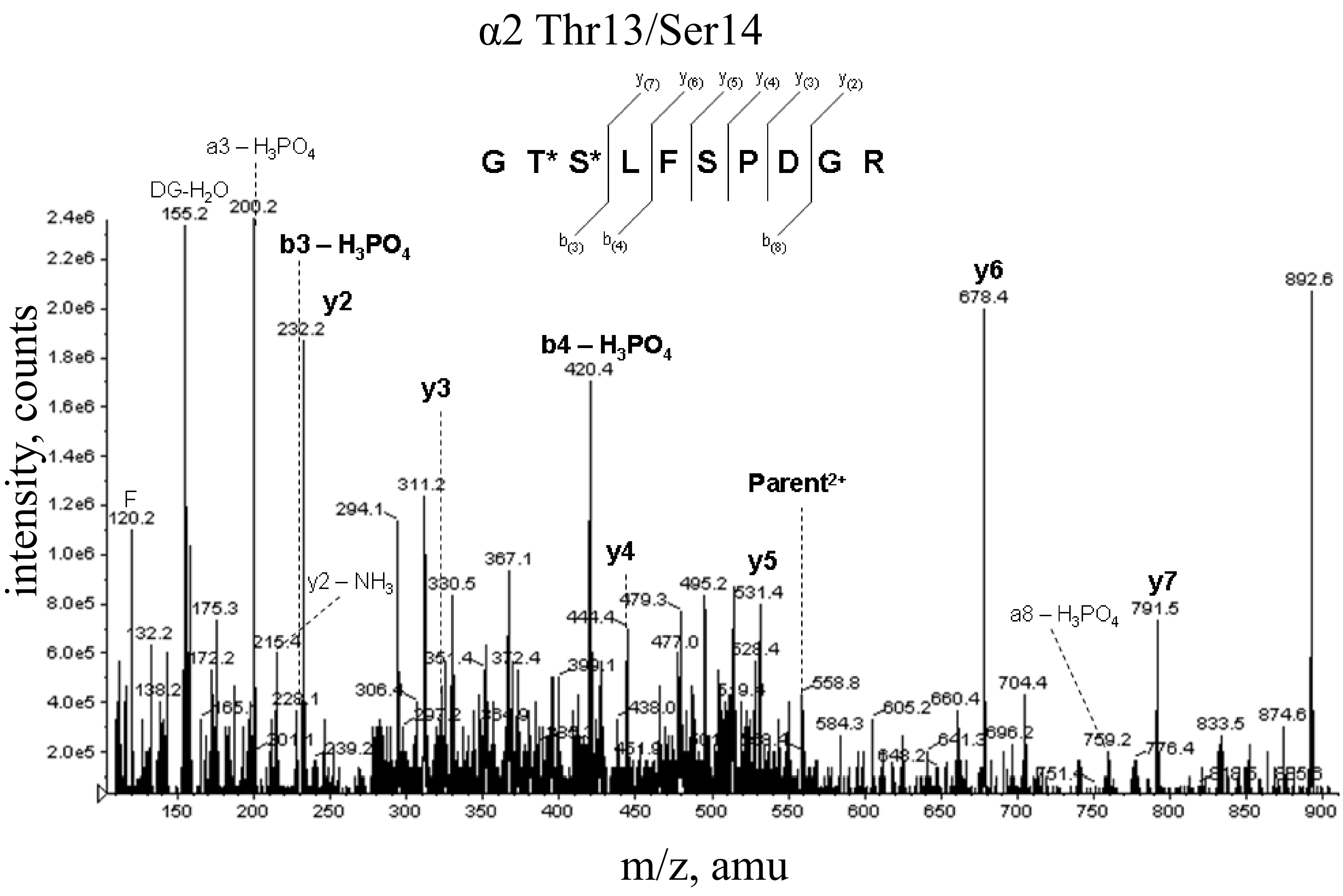

Suppl. Fig. 3. PanA is phosphorylated at Ser340.

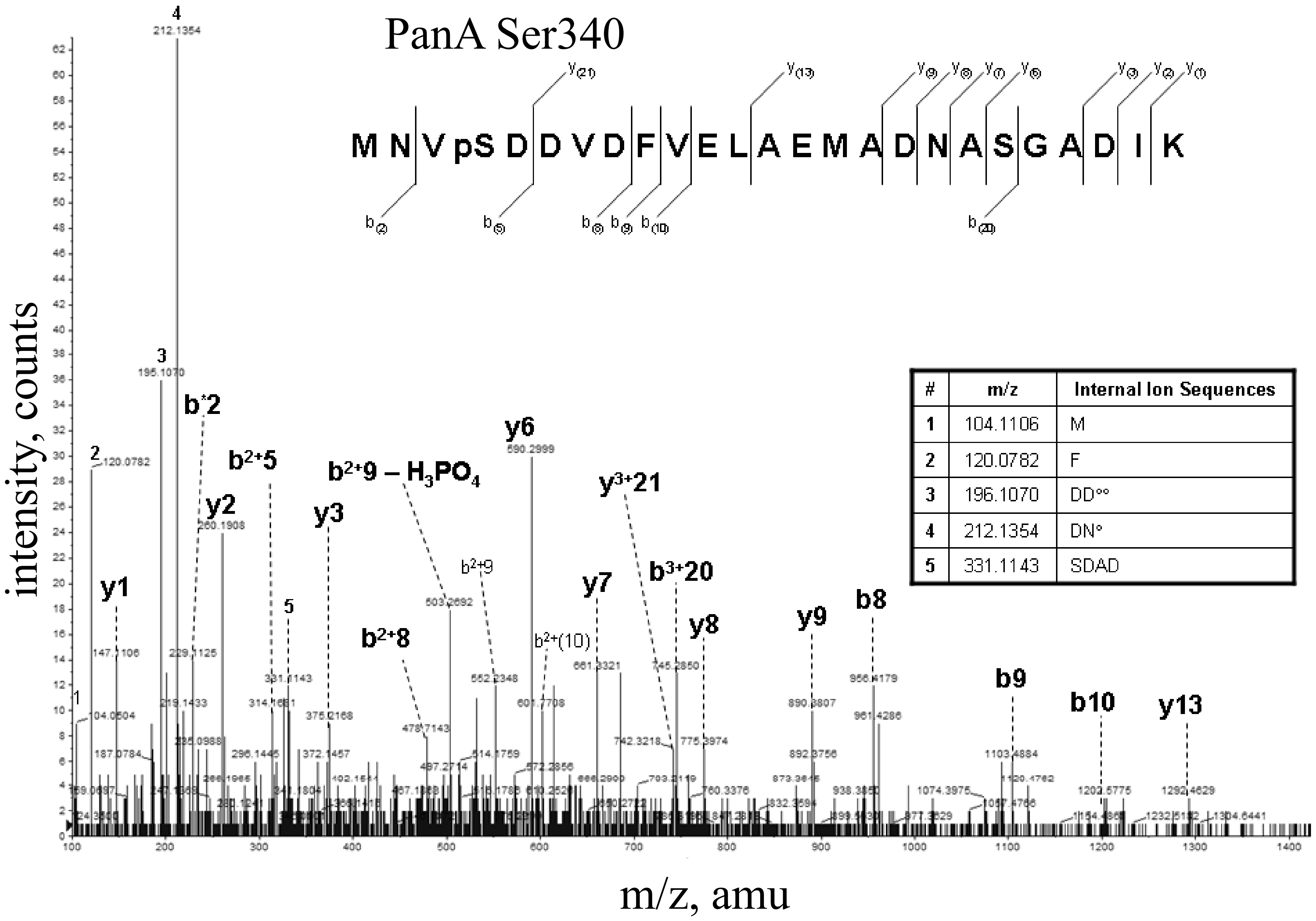

Suppl. Fig. 4. α1 is methylesterified on acidic residues.

A)

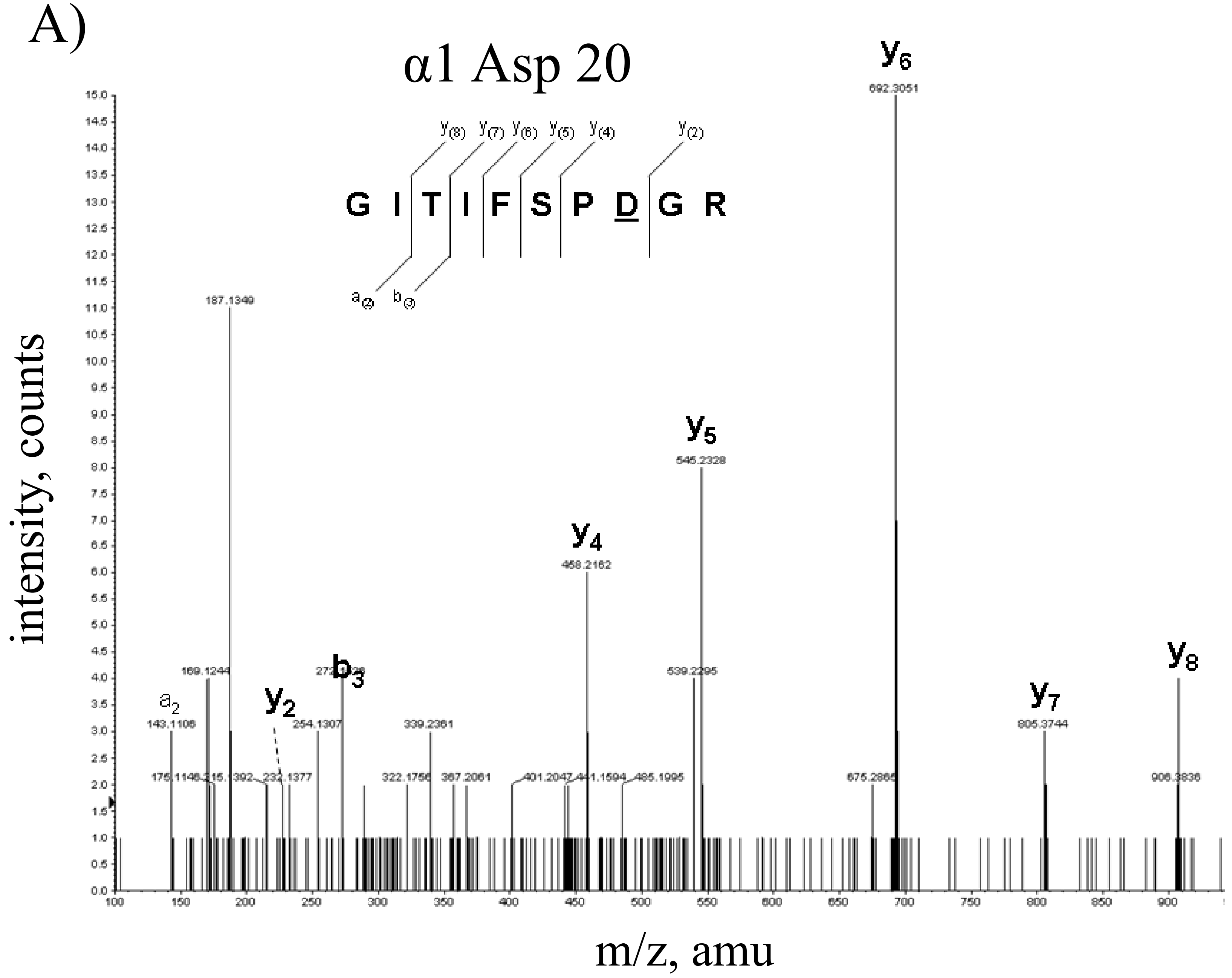

Suppl. Fig. 4. α1 is methylesterified on acidic residues.

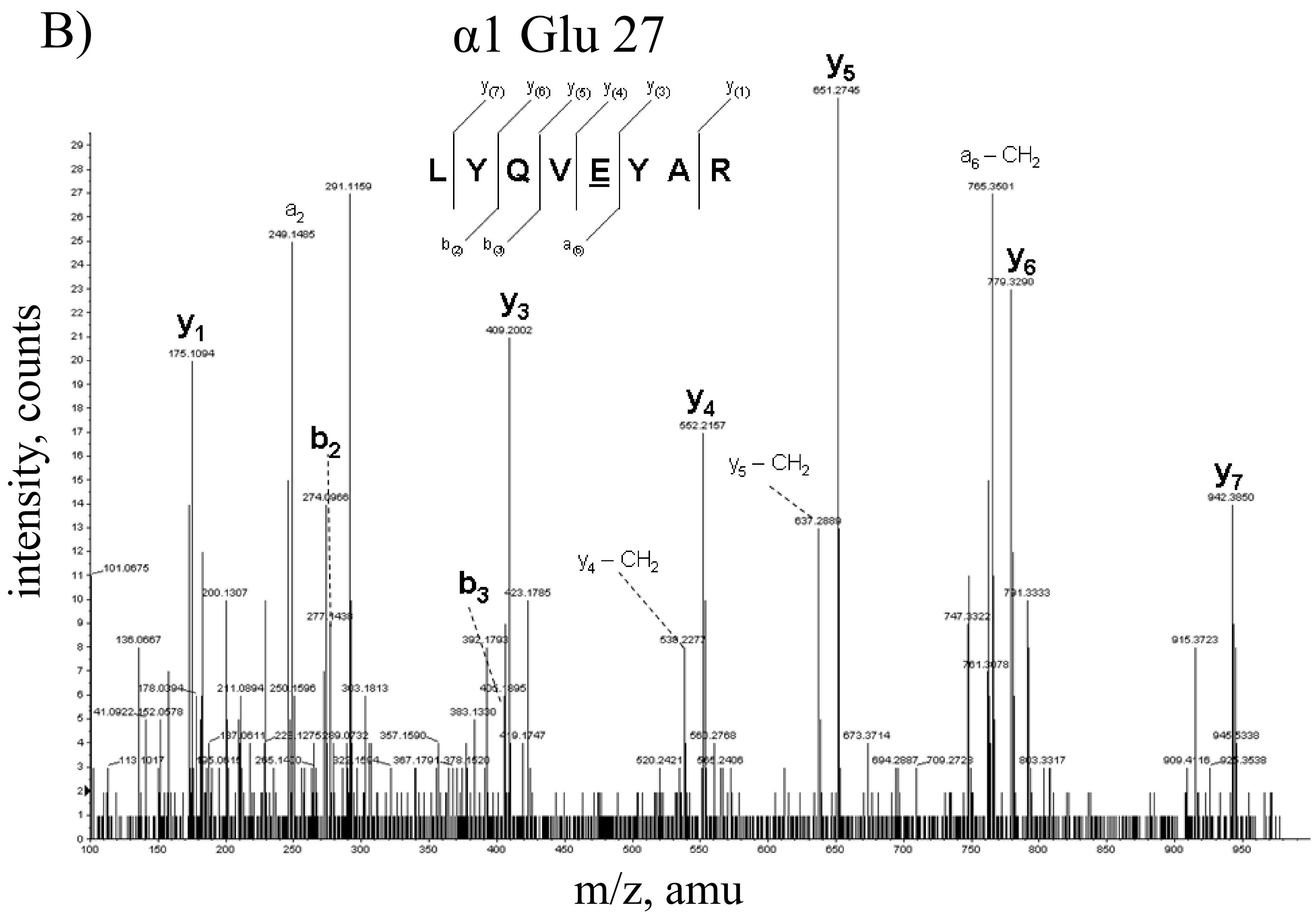

Suppl. Fig. 4. α1 is methylesterified on acidic residues.

C)

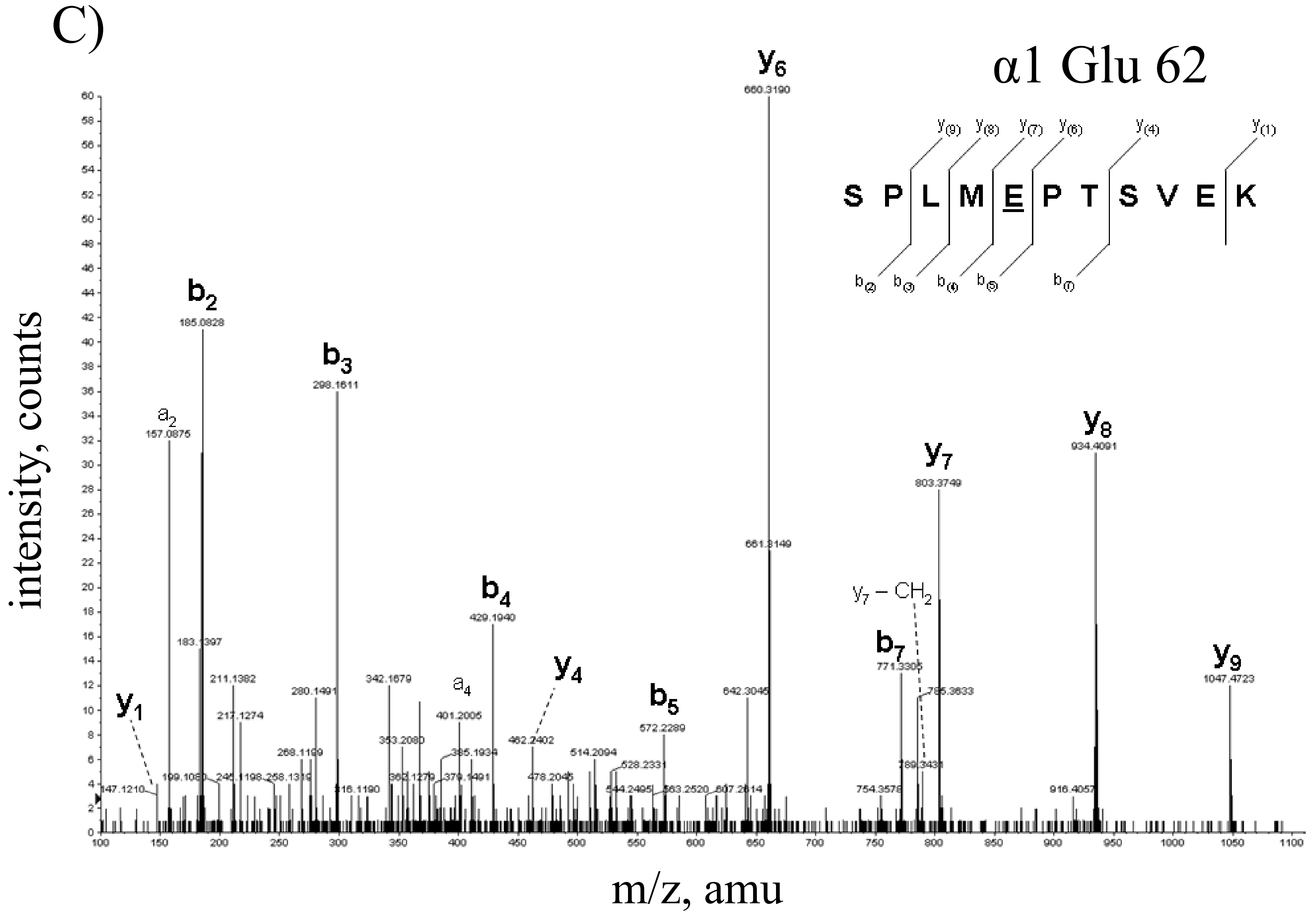

Suppl. Fig. 4. α1 is methylesterified on acidic residues.

D)

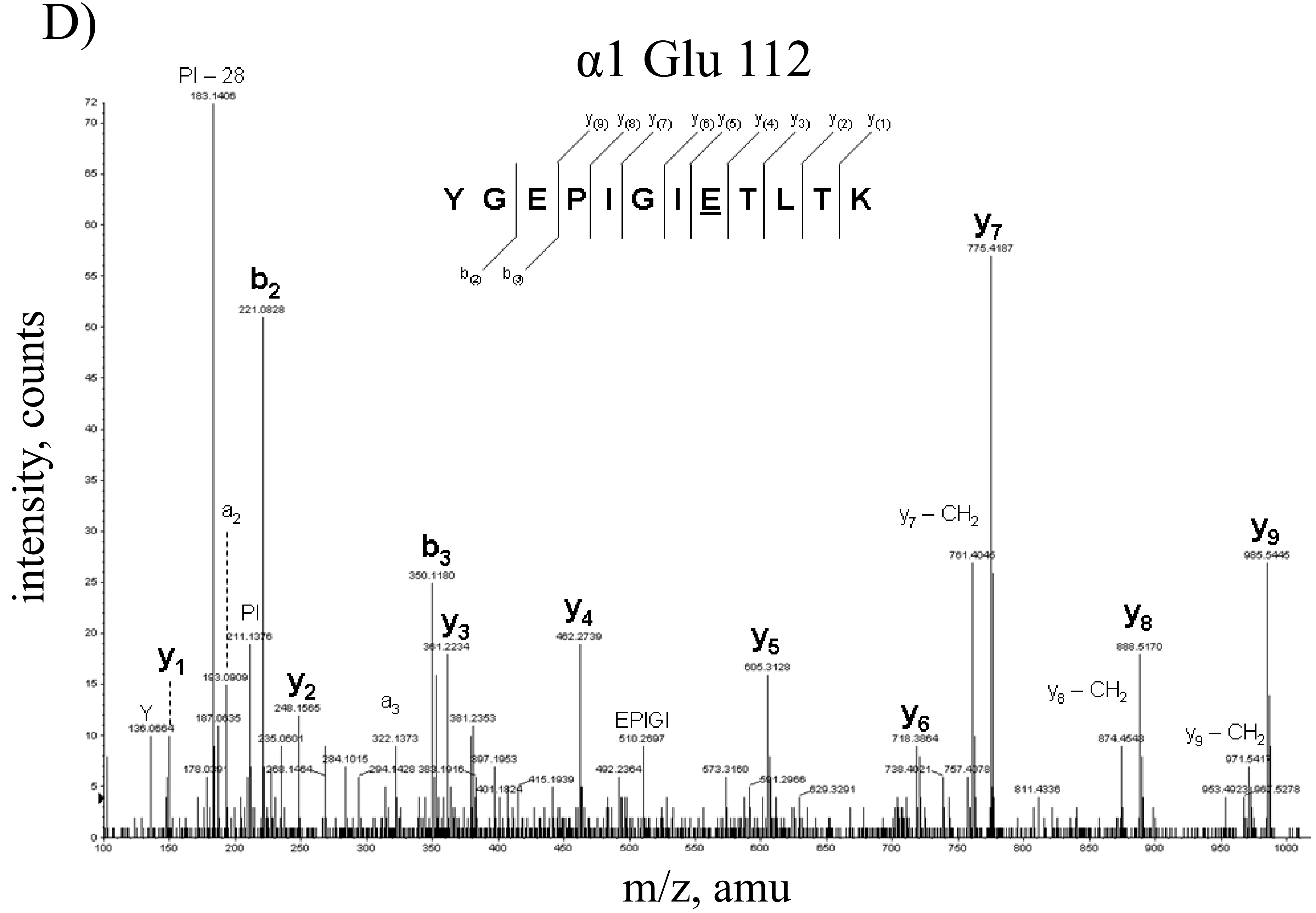

Suppl. Fig. 4. α1 is methylesterified on acidic residues.

E)

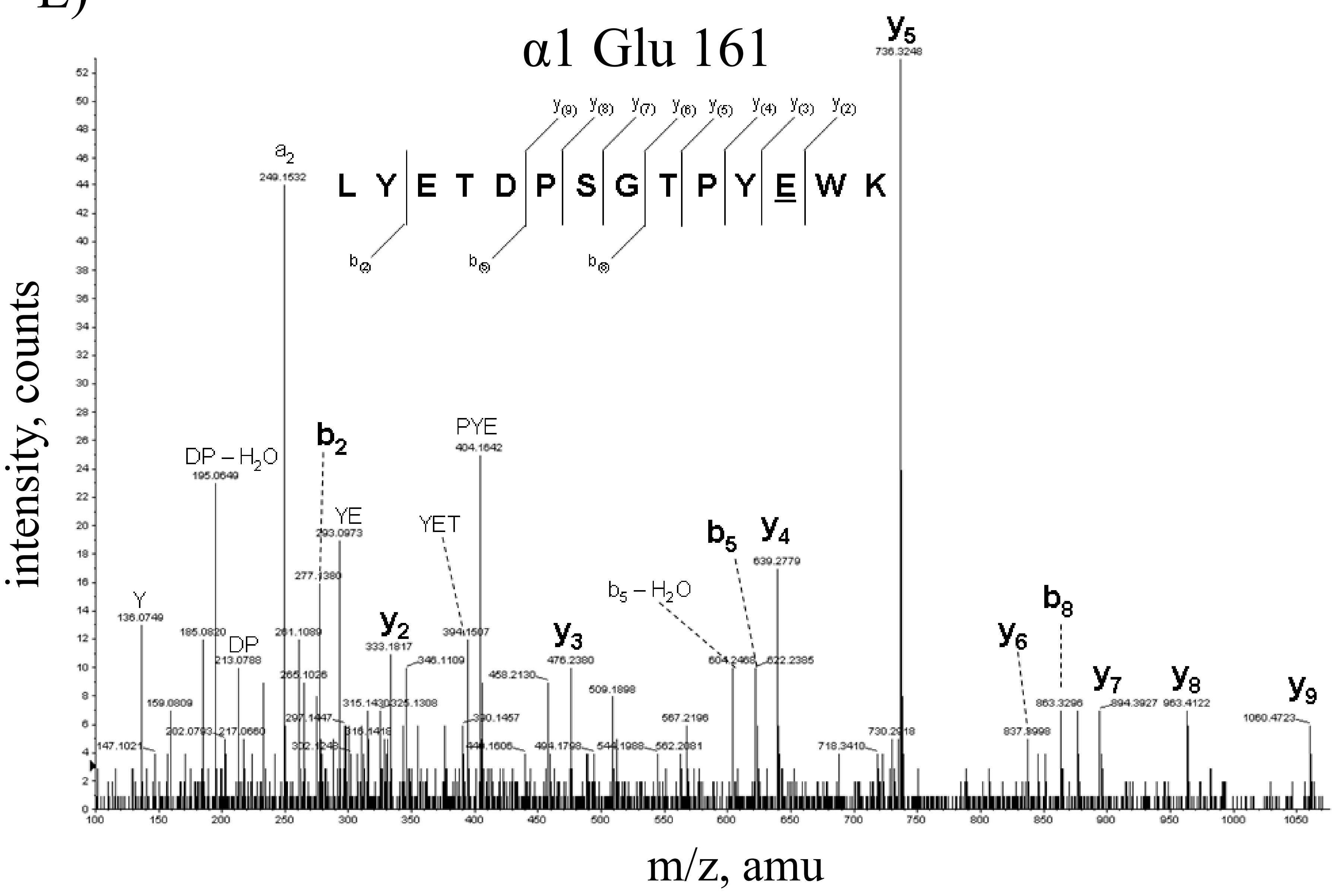

Supplement: Supplementary file 1 — Supplementary material includes a list of the strains and plasmids used in this study, oligonucleotide primer pairs used for site-directed mutagenesis, MS/MS-results that support the homogeneity of Rio1p kinase from H. volcanii, and mass spectra used to identify the sites of post-translational modification of H. volcanii proteasomes including the phosphosites α 1 Thr147, α 2 Thr13/Ser14 and PAN-A Ser340 as well as the methylesterification sites α 1 Asp20, Glu27, Glu62, Glu112 and Glu161. [file 481725.f1.pdf]
